# Immune Checkpoint PD‐L1 Modulates Retinal Microglial Activation to Alleviate Vascular Leakage in Choroidal Neovascularization via ERK

**DOI:** 10.1002/advs.202400747

**Published:** 2025-05-21

**Authors:** Yue Zou, Junliang Jiang, Yunqin Li, Xinyi Ding, Qiuping Tong, Ying Shi, Lei Xiao, Ling Chen

**Affiliations:** ^1^ Eye Institute and Department of Ophthalmology Eye & ENT Hospital NHC Key Laboratory of Myopia (Fudan University); Key Laboratory of Myopia Chinese Academy of Medical Sciences Shanghai Key Laboratory of Visual Impairment and Restoration Fudan University Shanghai 200031 China; ^2^ The State Key Laboratory of Medical Neurobiology and MOE Frontiers Center for Brain Science and the Institutes of Brain Science Fudan University Shanghai 200032 China; ^3^ Department of Ophthalmology Yunnan Eye Institute & Key Laboratory of Yunnan Province Yunnan Eye Disease Clinical Medical Center Affiliated Hospital of Yunnan University Yunnan University Kunming 650021 China; ^4^ Department of Orthopedics & Traumatology Affiliated Hospital of Yunnan University Yunnan University Kunming 650021 China

**Keywords:** microglia/macrophage, neovascular age‐related macular degeneration, neuroinflammation, PD‐L1

## Abstract

Neovascular age‐related macular degeneration (NVAMD) is a common retinal disease causing vision loss in the elderly. Neuroinflammation significantly contributes to NVAMD's etiology. This study explores the role of Programmed cell death ligand 1 (PD‐L1), an immune checkpoint (ICP) in microglia, known for limiting neuroinflammation in neurodegenerative diseases, and its potential function in NVAMD. This work finds increased PD‐L1 expression in retinal microglia following laser injury. PD‐L1 knockout (KO) or inhibitory PD‐L1 antibody treatment worsens vascular leakage and neoangiogenesis in a laser‐induced NVAMD mouse model, effects reversible by microglia depletion with PLX5622. This study underscores that choroidal neovascularization (CNV) may be regulated by multiple mechanisms, with PD‐L1 modulation representing one of these pathways. Blocking PD‐L1 elevated proinflammatory factors and p‐ERK levels, indicating microglial overactivation in NVAMD. Conversely, enhancing PD‐L1 signaling reduced neuroinflammation and neovascularization via ERK. These findings highlight PD‐L1's role in neoangiogenesis and neuroinflammation in NVAMD, suggesting its potential as a target for immunomodulatory treatment in NVAMD.

## Graphical abstract

PD‐L1 limits neuroinflammation in neovascular AMD by modulating microglial activation. Its deficiency exacerbates vascular leakage and choroidal neovascularization (CNV) via ERK signaling, while enhancing PD‐L1 reduces inflammation and neovascularization. Targeting PD‐L1 may offer a novel immunomodulatory strategy for NVAMD.

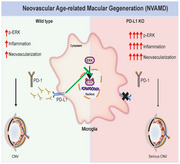

## 1 Introduction

Age‐related macular degeneration (AMD) is a chronic, progressive ocular disorder primarily affecting the central vision, making it a leading cause of irreversible blindness in the elderly population worldwide.^[^ ^1^, ^2^, ^3^ ^]^ It was estimated that by 2020, approximately 196 million people worldwide were affected by AMD, and this number is projected to reach 288 million by 2040.^[^ ^1^, ^4^, ^5^ ^]^ AMD predominantly affects individuals over the age of 50 and significantly impairs activities such as reading, driving, and facial recognition, thus diminishing patients' quality of life.^[^ ^6^ ^]^ Furthermore, AMD increases the risk of falls, depression, and loss of independence, making it a growing public health concern.^[^ ^7^, ^8^, ^9^ ^]^

Currently, anti‐vascular endothelial growth factor (anti‐VEGF) therapy is the primary therapeutic approach for the neovascular age‐related macular degeneration (NVAMD).^[^ ^3^, ^10^ ^]^ Nevertheless, anti‐VEGF therapy has some limitations, such as the economic burden, complications arising from frequent intravitreal injections and the treatment resistance.^[^ ^11^ ^]^ Significantly, approximately one‐third of patients fail to benefit from anti‐VEGF therapy owing to the macular fibrosis or atrophy.^[7]^ Furthermore, prolonged administration of anti‐VEGF agents may potentially result in neuronal damage.^[^ ^12^ ^]^ Given the limitation of anti‐VEGF therapies, there is a pressing need to explore the pathogenesis of NVAMD and the novel alternative therapies.

Genetic aberrations associated with innate immunity have been empirically established as contributory factors in increasing susceptibility to NVAMD.^[^ ^13^, ^14^, ^15^ ^]^ Studies from both NVAMD patients and animal models further provide evidence of perturbed innate immune homeostasis.^[^ ^16^, ^17^, ^18^ ^]^ Retinal microglia, the resident immune cells, regulate the tissue integrity in NVAMD.^[^ ^19^ ^]^ During the early stage of NVAMD, degeneration of the retinal pigment epithelium (RPE) and disruption of Bruch's membrane trigger the proliferation and migration of microglia to the injury site, which will facilitate tissue repair by releasing neuromodulators.^[^ ^20^ ^]^ However, neuroprotective role of the activated microglia is transient, and hyperactive microglia may leading to vascular leakage, which may contribute to choroidal neovascularization (CNV) progression.^[^ ^21^, ^22^ ^]^ Targeting retinal immune homeostasis and modulating microglial reactivity may be a potential strategy to mitigate and alleviate the progression of NVAMD.

Immune checkpoints (ICPs) play a pivotal role in regulating immune homeostasis, particularly in the neuroimmune and neuroinflammatory responses.^[^ ^23^ ^]^ While the bulk of research efforts have been dedicated to elucidate the functional intricacies of ICP molecules in cancer and peripheral immunity,^[^ ^24^, ^25^, ^26^, ^27^, ^28^ ^]^ some ICPs are also expressed in the central nervous system (CNS) cells, encompassing neurons,^[^ ^29^, ^30^ ^]^ astrocytes,^[^ ^23^, ^31^ ^]^ and microglia.^[^ ^32^, ^33^, ^34^ ^]^ ICPs are linked with the maintenance of CNS immune homeostasis, as well as the progression of neuroinflammatory and neurodegenerative diseases.^[^ ^33^, ^35^ ^]^ One of the prominent ICPs expressed in CNS is Programmed Cell Death Protein 1 (PD‐1),^[^ ^36^, ^37^ ^]^ accompanied by its ligands Programmed Cell Death lig 1 (PD‐L1)^[^ ^38^ ^]^ and PD‐L2.^[^ ^39^ ^]^ PD‐L2 is predominantly expressed in antigen‐presenting cells, whereas PD‐L1 has a broad expression.^[^ ^39^ ^]^ Clinical investigations reported the elevation of PD‐1 level in NVAMD patients^[^ ^40^ ^]^ and after block PD‐1/PD‐L1, NVAMD patients undergo unresponsive to anti‐VEGF treatments,^[^ ^41^ ^]^ which suggests the potential alterations of PD‐1/PD‐L1 pathway during the pathogenesis of NVAMD.

To investigate the role of retinal PD‐L1 in the progression of NVAMD, we established a laser‐induced CNV mouse model.^[^ ^42^ ^]^ Our results demonstrated a significant upregulation of PD‐L1 expression in retinal microglia within this model. PD‐L1 blockade led to a pronounced exacerbation of retinal neuroinflammation and pathological angiogenesis, highlighting its regulatory role in immune modulation through intrinsic microglial signaling pathways. These findings provide critical insights into the potential therapeutic application of ICP regulators for improving NVAMD treatment.

## 2 Results

### 2.1 Retinal PD‐L1 Expression Increased in Laser‐Induced CNV

**Figure 1:**
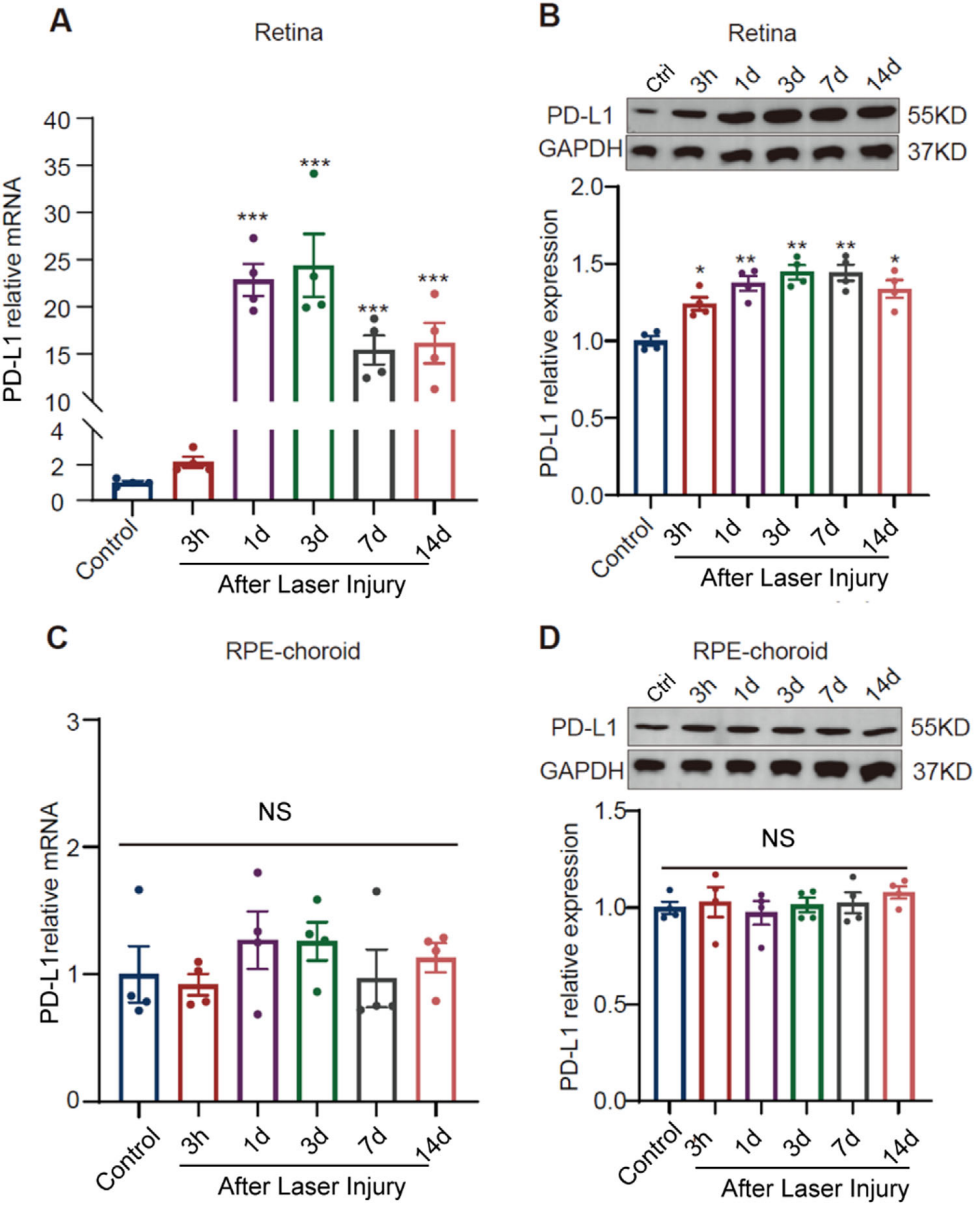
PD‐L1 expression in retina and retinal pigment epithelium (RPE) choroid in the laser‐induced choroidal neovascularization (CNV) mouse model. A) Statistical result of PD‐L1mRNA expression change in mouse retina at various time points: Control, 3 h post‐laser (3 h), 1‐, 3‐, 7‐, and 14‐days post‐laser (1, 3, 7, and 14 days). B) Western blot example images (top) and statistical result (bottom) of PD‐L1 protein level in the mouse retina at different time points. C) Same as (A), but for the RPE‐choroid tissue. D) Same as (B), but for the RPE‐choroid tissue (*n* = 4 mice for each group). Data are presented as mean ± SEM. ^*^ *p* < 0.05, ^**^ *p* < 0.01, ^***^ *p* < 0.001, compared to the Control group. One‐way ANOVA with Tukey's multiple comparisons post‐hoc test.

In the CNS, neuroinflammation upregulates PD‐L1 (encoded by the *Cd274* gene) to maintain immune homeostasis.^[^ ^43^ ^]^ However, it remains unclear whether retinal PD‐L1 expression is altered during CNV formation. To investigate this, we measured retinal PD‐L1 mRNA and protein levels in mice following laser injury. Compared to controls, PD‐L1 mRNA level increased approximately 22‐fold at 1‐day post‐laser photocoagulation, peaking at 3 days and gradually declining, yet remaining significantly elevated (**Figure** **1**A). Correspondingly, retinal PD‐L1 protein expression also rose as early as 3 h post‐laser treatment and persisted for over 14 days (Figure 1B). Since PD‐L1 is also expressed in RPE,^[^ ^44^, ^45^ ^]^ we investigated the change of PD‐L1 expression within the RPE‐choroid tissue. Our results showed no significant changes in PD‐L1 mRNA (Figure 1C) and protein (Figure 1D) after laser photocoagulation. These findings indicate that PD‐L1 expression in the retina is markedly elevated during laser‐induced CNV formation.

### 2.2 PD‐L1 Knockout or Blockade Increase Vascular Permeability and Neovascularization in CNV

Given the significant increase of retinal PD‐L1 level following the laser injury, we explored the correlation between PD‐L1 expression and the initiation of CNV by changing PD‐L1. Firstly, we used a PD‐L1 knockout (KO) mouse line, and the successful deletion of PD‐L1 was confirmed by genomic PCR of mouse tail DNA samples and WB analyses of retina‐RPE‐choroidal lysates (Figure S1A,B, Supporting Information).

The formation of CNV in mice typically peaks on day 7 after laser injury.^[^ ^42^, ^46^ ^]^ Therefore, we selected this time point to assess the severity of CNV in each group of mice, primarily evaluating two parameters: vascular permeability and the extent of neovascularization.^[^ ^46^ ^]^ In this study, we used fluorescein fundus angiography (FFA) and optical coherence tomography (OCT) to observe vascular leakage and subretinal fluid accumulation, which were used to assess vascular permeability.^[^ ^47^ ^]^ We quantified CNV thickness using OCT and determined its surface area through IB4 staining of RPE‐choroid flat mounts to comprehensively evaluate the extent of CNV.^[^ ^42^ ^]^

**Figure 2:**
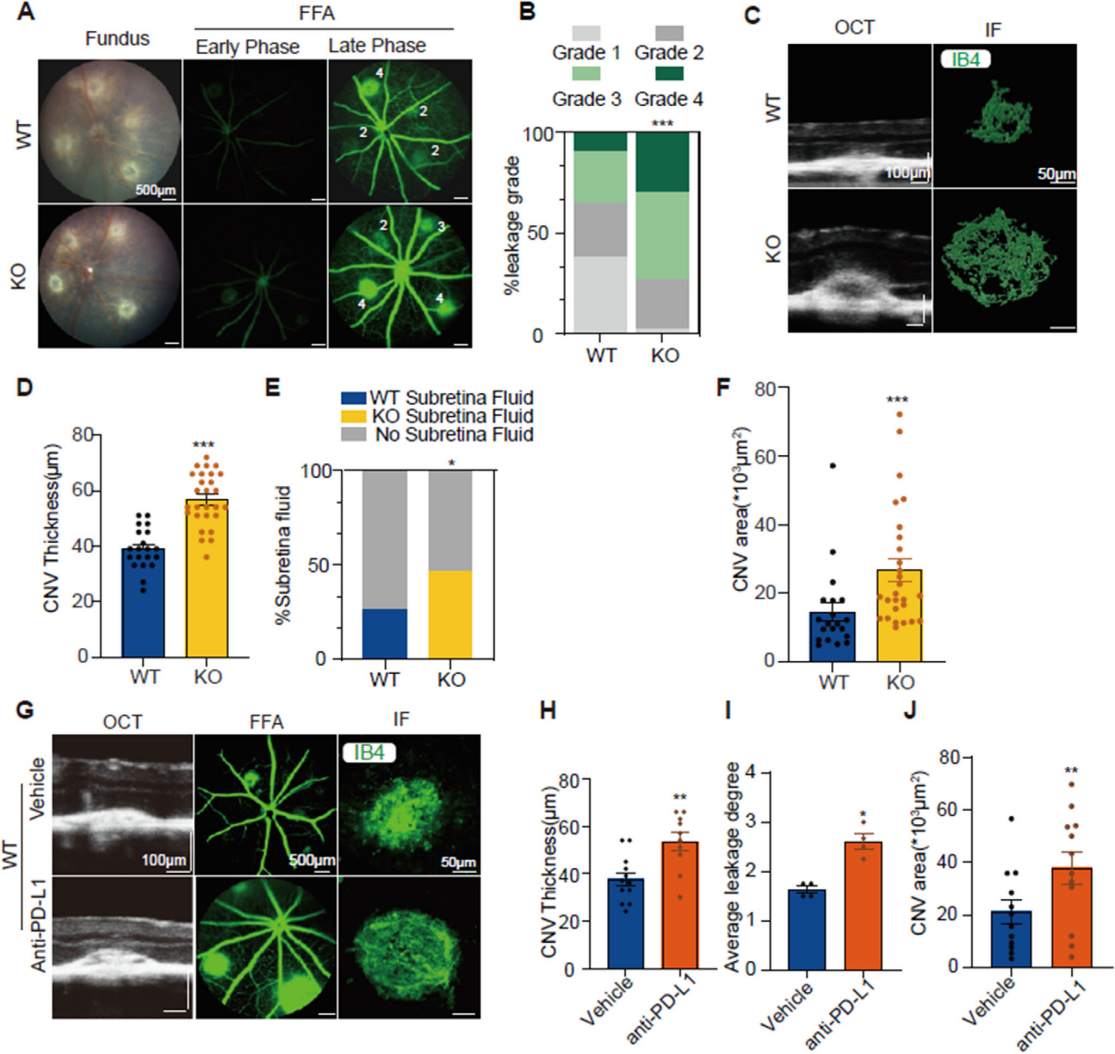
PD‐L1 knockout (KO) or blockade increase vascular permeability and neovascularization in choroidal neovascularization (CNV). A) Left panels display the fundus images of WT and KO mice at 7 days post‐laser injury. Middle and right panels present fluorescein fundus angiography (FFA) images of WT and KO mice at two distinct time points (early and late phases) at 7 days after laser injury, respectively. B) Summaries of the FFA grade scores of each laser spot (*n* = 42 and 37 laser spots from WT and KO mice, respectively). C) Left panels show the examples of optical coherence tomography (OCT) scan, and right panels show the laser‐induced CNV stained with IB4 in retinal pigment epithelium (RPE)/choroidal flat mounts at 7 d after laser injury from WT and KO mice. D) Quantification of the laser‐induced CNV thickness and E) percentages of subretinal fluids after laser injury (*n* = 20 and 27 laser spots from WT and KO mice, respectively). F) Quantification of the laser‐induced CNV area in RPE/choroidal flat mounts (*n* = 7 WT mice and 10 KO mice). G) Left panels show the examples of OCT scan, middle panels show FFA images and right panels show images of IB4 staining in RPE/choroidal flat mounts at 7 days after laser injury with anti‐PD‐L1 antibody or vehicle intravitreal injections. H–J) Quantifications of the laser‐induced CNV thickness (J), percentages of subretinal fluids (K), and laser‐induced CNV area (L) (*n* = 4 mice per group). Data are presented as mean ± SEM. Unpaired *t*‐test for CNV thickness and CNV area in (D,F,H,J). ^*^ *p* < 0.05, ^**^ *p* < 0.01, ^***^ *p* < 0.001 compared to the WT‐CNV group or vehicle group. Mann–Whitney test for average FFA grade scores in (I), ^*^ *p* < 0.05, ^**^ *p* < 0.01, ^***^ *p* < 0.001 compared to the vehicle group. Chi‐square test for FFA grade scores in (B) and percentages of subretinal fluids in (E). ^*^ *p* < 0.05, ^**^ *p* < 0.01, ^***^ *p* < 0.001 compared to the WT‐CNV group. Source data are provided as a Source Data file.

The experimental results showed that, on day 7 after laser modeling, PD‐L1 KO mice exhibited more extensive and severe vascular leakage during both early and late phases of FFA, with leakage scores significantly higher than those in WT mice (**Figure** **2**A,B). Consistent with the FFA results, OCT detection revealed that the proportion of subretinal fluid at the laser injury sites in PD‐L1 KO mice was significantly higher than in WT mice (38.5% versus 21.8%, respectively) (Figure 2C). Further OCT evaluation of CNV thickness showed that PD‐L1 KO mice had significantly thicker CNV compared to WT mice (Figure 2D). The IB4 staining of RPE‐choroid flat mounts also confirmed that the CNV surface area in PD‐L1 KO mice was markedly larger than in WT mice (Figure 2C,F). These results suggest that PD‐L1 gene KO leads to increased vascular permeability and neovascularization in mice after laser injury, resulting in more severe CNV.

To exclude the potential interference of systemic PD‐L1 KO on ocular development and overall systemic effects, we injected a PD‐L1 blocking antibody (anti‐PD‐L1) into the vitreous of WT mice to assess the impact of locally blocking intraocular PD‐L1 signaling on CNV formation. The results showed that, similar to the PD‐L1 KO group, mice that received intravitreal injections of anti‐PD‐L1 exhibited significantly higher fluorescence leakage scores compared to the control vehicle group on day 7 after laser‐induced CNV formation (Figure 2G,I). Additionally, both the thickness and area of neovascularization were significantly increased following anti‐PD‐L1 injection (Figure 2G,H,J). These findings suggest that local blockade of PD‐L1 within the eye has effects similar to systemic PD‐L1 KO, leading to increased vascular permeability and neovascularization area, resulting in more severe CNV in the model mice.

### 2.3 Activation of PD‐L1 Ameliorates CNV in Mice

Previous experimental results demonstrated that blocking PD‐L1 exacerbates CNV formation in mice. This suggests that the upregulation of PD‐L1 observed during CNV might represent a protective anti‐inflammatory response, similar to its role in other nervous system diseases.^[^ ^43^ ^]^ However, whether further activation of PD‐L1 could improve CNV pathology remains unclear. Studies have shown that soluble PD‐1 can bind to PD‐L1, triggering downstream signaling cascades.^[^ ^48^ ^]^ In neuroinflammatory conditions such as multiple sclerosis, PD‐L1 expressed on immune cells interacts with PD‐1 to transmit inhibitory signals, playing a crucial role in maintaining immune homeostasis and suppressing inflammation.^[^ ^49^ ^]^ We hypothesized that exogenous PD‐1 protein, by binding to PD‐L1, might activate downstream PD‐L1 signaling and further inhibit CNV formation in the CNV mouse model.

**Figure 3:**
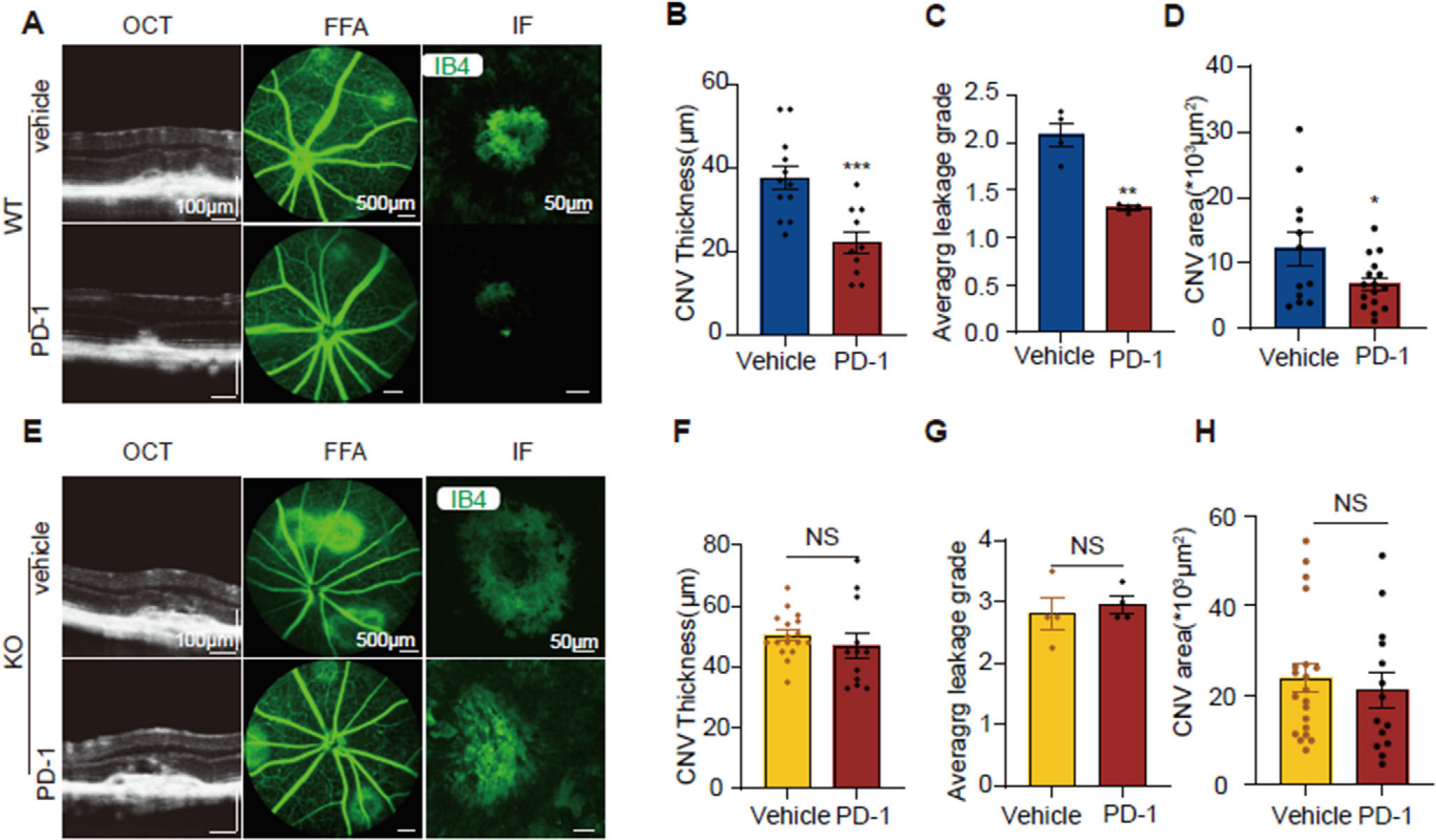
Activation of PD‐L1 ameliorates choroidal neovascularization (CNV) in mice. A) Left panels show the examples of optical coherence tomography (OCT) scan, middle panels show the fundus fluorescein angiography (FFA) example images, and right panels show the IB4 staining in retinal pigment epithelium (RPE)/choroidal flat mounts in WT mice after intravitreal injection of PD‐1 or vehicle and 7 days after laser injury. B–D) Quantifications of the laser‐induced CNV thickness (B), percentage of subretinal fluids (C), and laser‐induced CNV area (D) (*n* = 4 mice per group). E) Same as (A), but for the PD‐L1 KO mice. F–G) Same as (B–D), but for the PD‐L1 KO mice (*n* = 4 mice per group). Data are presented as mean ± SEM. Unpaired *t*‐test for CNV thickness in (B) and (F), average leakage degree in (C) and (G), CNV area in (D) and (H). ^^^ *p* < 0.05, ^^^^ *p* < 0.01, and ^^^^^ *p* < 0.001, compared to the control group. ^*^ *p* < 0.05, ^**^ *p* < 0.01, and ^***^ *p* < 0.001, compared to the CNV + vehicle group. Source data are provided as a Source Data file.

To test this hypothesis, we injected PD‐1 protein or an equal volume of control vehicle into the vitreous of WT and PD‐L1 KO mice following laser photocoagulation. The FFA results showed that intravitreal injection of PD‐1 significantly suppressed vascular leakage in WT mice compared to control vehicle (**Figure** **3**A,C). OCT examinations and IB4 staining of RPE‐choroid flat mounts further demonstrated that PD‐1 injection significantly reduced CNV thickness and area in WT mice (Figure 3A,B,D). However, in PD‐L1 KO mice, intravitreal injection of PD‐1 protein did not significantly reduce vascular leakage, nor did it decrease CNV area or thickness compared to vehicle injection (Figure 3E–H). These findings indicate that in the CNV mouse model, intravitreal administration of exogenous PD‐1 targets PD‐L1 and enhances its protective effects, thereby reducing vascular leakage and pathological neovascularization.

### 2.4 PD‐L1 Deficiency Exacerbates CNV by Enhancing Microglial Activation and Neuroinflammation

**Figure 4:**
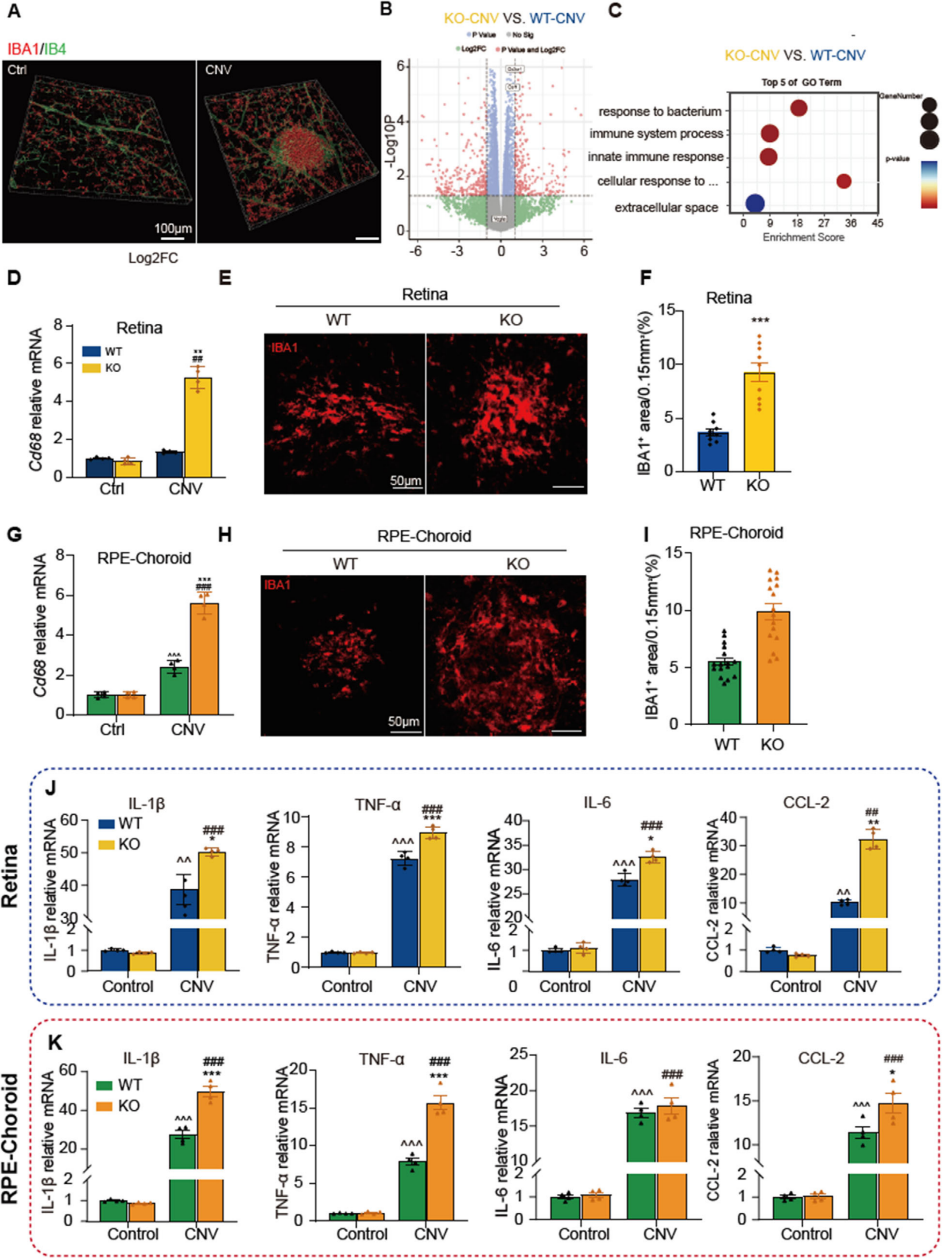
PD‐L1 knockout (KO) promotes inflammatory activation of microglia in choroidal neovascularization (CNV) mice. A) IB4 (green) and IBA1(red) staining and 3D reconstruction of retinal flat mounts from control group (Left) and CNV model mice (Right). IBA1+ microglia were clustered at the laser lesioned location. B) Volcano plot obtained from DESeq2 analysis of the retina in the laser‐induced CNV mouse model, comparing wild‐type CNV (WT‐CNV) and KO (KO‐CNV) mice at 3 days after laser injury (*n* = 4 mice per group). C) Gene Ontology (GO) functional enrichment analysis of the differential expression genes (DEGs). D) Expression levels of CD68 mRNA in the retinas of WT and PD‐L1 KO mice from control and laser‐treated groups, assessed 3 days after laser injury. E) Representative images of IBA1+ cells at the laser lesion sites in the retina. F) Quantitative analysis of the IBA1+ area at the laser lesion sites in the retina. G–I) Corresponding analyses as described in (D–F), performed on retinal pigment epithelium (RPE) choroid tissue samples. J) Expression levels of IL‐1β, IL‐6, TNF‐α, and CCL‐2 mRNA in the retinas of WT and PD‐L1 KO mice from control and CNV groups, evaluated 3 days after laser‐induced injury. K) Corresponding analyses for IL‐1β, IL‐6, TNF‐α, and CCL‐2 mRNA expression in RPE‐choroid tissue samples. Data are presented as mean ± SEM, with *n* = 4 mice per group. Statistical significance is denoted as follows: ^^^ *p* < 0.05, ^^^^ *p* < 0.01, ^^^^^ *p* < 0.001 (compared to the WT‐control group); ^*^ *p* < 0.05, ^**^ *p* < 0.01, ^***^ *p* < 0.001 (compared to the WT‐CNV group); and ^#^ *p* < 0.05, ^##^ *p* < 0.01, ^###^ *p* < 0.001 (compared to the KO‐control group). Statistical analysis was performed using one‐way ANOVA followed by Tukey's multiple comparisons test.

Prior studies suggested a strong correlation between vascular permeability and the extent of inflammation.^[^ ^46^, ^50^ ^]^ The pronounced vascular leakage we observed in PD‐L1 KO mice indicates the elevation of retinal neuroinflammation after CNV. Inflammation in CNV is largely mediated by microglia and macrophages,^[^ ^51^ ^]^ which also express PD‐L1.^[^ ^34^ ^]^ In conditions like multiple sclerosis^[^ ^49^ ^]^ and spinal cord injury,^[^ ^43^ ^]^ blocking PD‐L1 in these cells worsens their activation and neuroinflammation. Our immunofluorescence (IF) and 3D reconstruction analyses further revealed a marked increase in IBA1+ cells, forming distinct clusters at the laser‐induced lesion site (**Figure** **4**A). Therefore, we hypothesized that PD‐L1 blockade may aggravate CNV by enhancing microglia/macrophage‐mediated inflammation. To test this hypothesis, we designed an RNA sequencing (RNA‐seq) experiment on retinal tissues from PD‐L1 KO and WT mice at the peak of microglial activation, which previous studies have identified as day 3 post‐laser.^[^ ^46^, ^52^ ^]^ Our results also confirmed this timing, showing substantial microglia accumulation at the laser sites on day 3 post‐laser (Figure S1C,D, Supporting Information). RNA‐seq analysis revealed significant gene expression differences between PD‐L1 KO and WT mice. Specifically, 10 017 genes were upregulated and 10 610 were downregulated in PD‐L1 KO mice compared to WT. Notably, genes linked to microglial activation, such as *Csf1* and *Cx3cr1*,^[^ ^53^, ^54^ ^]^ were significantly upregulated in PD‐L1 KO mice (Figure 4B). Gene Ontology (GO) enrichment analyses (Figure 4C) revealed a similar enrichment pattern, with differentially expressed genes involved in innate immunity and inflammation pathways. Top 5 Key terms included “response to bacterium” “immune system process,” “innate immune response,” “cellular response to interferon‐beta,” and “extracellular space.” These RNA‐seq results suggest that PD‐L1 deficiency may influence microglial activation, potentially driving the exacerbation of CNV.

To further validate this hypothesis, we assessed microglial/macrophage activation in the retinas of PD‐L1 KO and WT mice by measuring CD68 expression and IBA1+ cell numbers.^[^ ^55^, ^56^ ^]^ The results showed a significant increase in retinal *CD68* mRNA levels in WT mice on day 3 post‐laser, with an even greater elevation in PD‐L1 KO mice (Figure 4D). Immunostaining revealed substantial IBA1+ cell accumulation at laser sites in both groups, with significantly more IBA1+ cells in PD‐L1 KO mice (Figure 4E,F). A similar trend was observed in the RPE‐choroid tissue (Figure 4G–I).

Given that microglia and macrophages are major sources of proinflammatory cytokines in the retina and RPE‐choroid,^[^ ^57^, ^58^ ^]^ we compared cytokine expression levels in these tissues. On day 3 post‐laser, retinal and RPE‐choroid tissues showed significantly increased mRNA levels of *IL‐1β*, *IL‐6*, *TNF‐α*, and *CCL‐2*. In PD‐L1 KO mice, the upregulation of these cytokines was more pronounced (Figure 4J,K). These findings suggest that PD‐L1 plays a critical role in limiting inflammation in CNV, and its deficiency exacerbates CNV by enhancing microglial activation and neuroinflammation. In summary, these data suggest the expression of PD‐L1 in retinal microglial cells is increased after laser injury, and KO of PD‐L1 will promote retinal microglial cell activation following laser injury, which may exacerbate vascular leakage and neovascular lesions by elevating the release of proinflammatory neurotoxic factors.

An intriguing observation pertains to the mRNA expression levels of VEGF, a pivotal factor in promoting neovascularization.^[^ ^59^, ^60^ ^]^ VEGF mRNA levels in the RPE‐choroid of PD‐L1 KO mice were significantly elevated compared to those in WT mice, whereas no significant differences were observed between WT and PD‐L1 KO groups in the retina (Figure S2A,C, Supporting Information), consistent with RNA‐seq results (Figure 4B). These findings indicate that PD‐L1 deficiency exacerbates inflammation in the retina and RPE‐choroid following laser‐induced CNV and is associated with increased VEGF mRNA expression in the choroid. However, the absence of PD‐L1 does not appear to significantly influence VEGF mRNA levels in the retina.

To further explore the underlying mechanism, we examined whether PD‐1‐induced PD‐L1 activation attenuates microglia/macrophage‐mediated inflammation. On the third day post‐laser injury, intravitreal PD‐1 injection in WT mice significantly reduced CD68 mRNA expression in the retina and RPE‐choroid (Figure S3A,G, Supporting Information), indicating effective suppression of microglia/macrophage activation. Additionally, in the CNV model, PD‐1 injection decreased the mRNA levels of inflammatory cytokines and chemokines, including IL‐1β, TNF‐α, IL‐6, and CCL‐2, in the retina and RPE‐choroid of WT mice (Figure 3B–E,H–K, Supporting Information). However, the influence of PD‐1 on VEGF mRNA levels in both the retina and RPE‐choroid is not statistically significant (Figure S2B,D, Supporting Information).

In conclusion, these results highlight the crucial role of PD‐L1 in moderating neuroinflammation and vascular responses in CNV. Pronounced vascular leakage in PD‐L1 KO mice, accompanied by increased microglial activation and elevated inflammatory cytokines, underscores PD‐L1's potential as a therapeutic target for controlling inflammation and preventing the progression of CNV.

### 2.5 Upregulation of PD‐L1 in Microglia/Macrophages Directly Modulates Microglial Inflammatory Responses

Studies have shown that PD‐L1 is expressed in microglia/macrophages and upregulated in response to inflammatory stimuli, transmitting inhibitory signals that prevent excessive activation of these cells and helping to regulate neuroinflammation.^[^ ^49^, ^61^ ^]^ However, it remains unclear whether PD‐L1 influences microglial function through a similar mechanism in CNV. Additionally, the expression of PD‐L1 in retinal and RPE‐choroid microglia/macrophages has not been thoroughly investigated. To address this, we first used IF staining to assess PD‐L1 expression in microglia/macrophages in both retinal and RPE‐choroid tissues.

**Figure 5:**
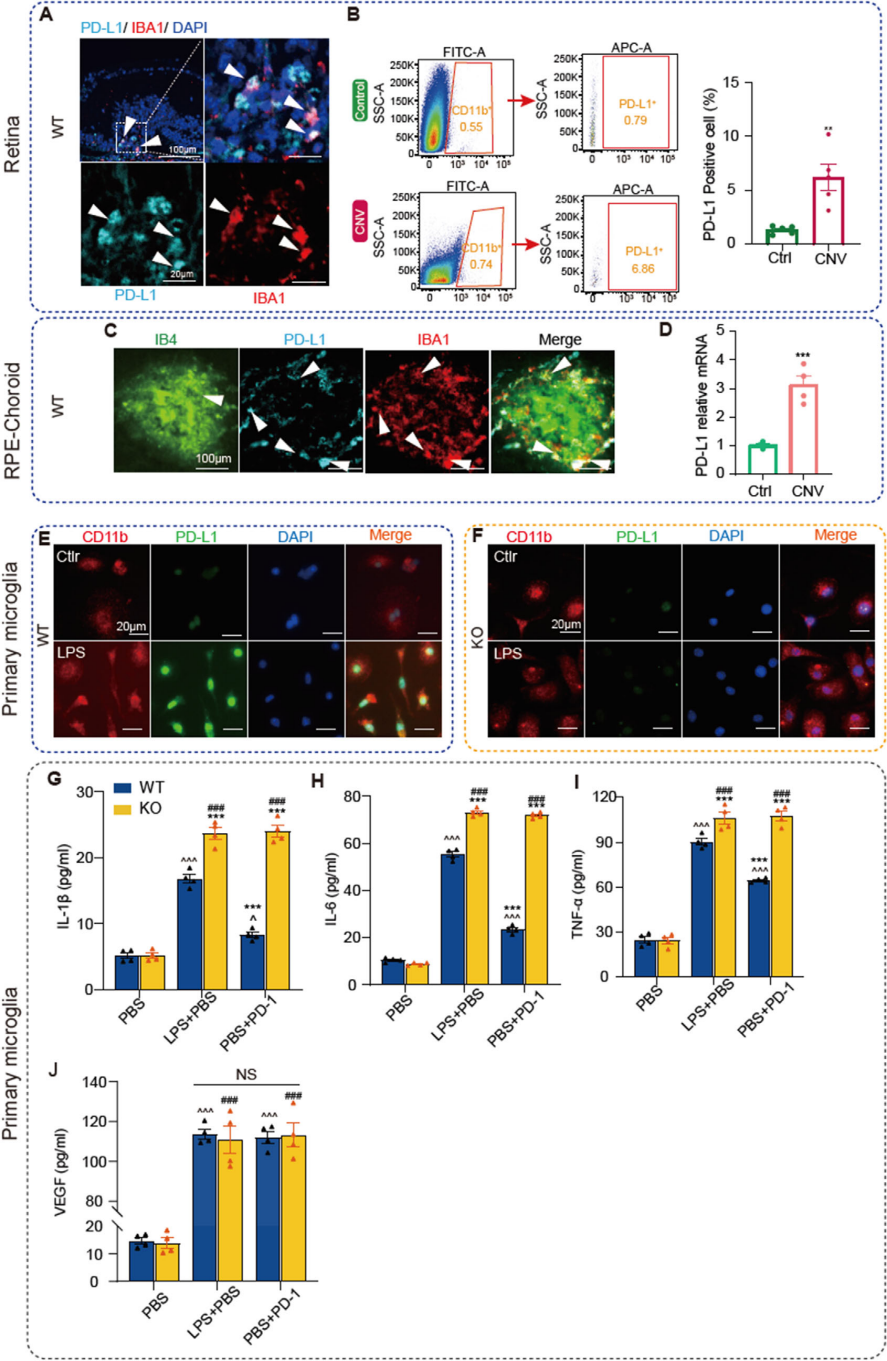
Upregulation of PD‐L1 in microglia/macrophages directly modulates microglial inflammatory responses. A) Immunofluorescence (IF) staining of the laser injured retina from wild‐type (WT) mouse at 3 days after laser injury with anti‐PD‐L1 (cyan) and anti‐IBA1 (red) antibodies. B) Left panels show the gating strategy for flow cytometric analysis of microglia cells in the retina. Microglia cells were identified by CD11b expression. Middle panels show the flow plots of PD‐L1+ microglia in retinas from control group and choroidal neovascularization (CNV) model mice. Right panels show percentages of PD‐L1+ microglia in control group and CNV model mice (*n* = 4 samples from control group and CNV model group). C) IF staining of the laser injured retinal pigment epithelium (RPE) choroid from WT mouse at 3 days after laser injury with anti‐PD‐L1 (cyan) and anti‐IBA1 (red) antibodies. D) Quantitative analysis of PD‐L1 mRNA in CD11b+ cells derived from the retinal pigment epithelium (RPE) choroid of WT mice, comparing the control group and laser‐treated group 3 days after laser injury (*n* = 4 samples from control group and CNV model group). E) IF staining of the primary microglia from WT mice with anti‐PD‐L1 (green) and anti‐CD11b (red) antibodies. F) Same as (E), but for the primary microglia from PD‐L1 KO mice. G–J) PBS control group, LPS group, and LPS + PD‐1 group. Levels of proinflammatory cytokines and VEGF secretion in primary microglia. The cells were derived from either WT or PD‐L1 KO mice (*n* = 4 per group). Data are presented as mean ± SEM, ^*^ *p* < 0.05, ^**^ *p* < 0.01, ^***^ *p* < 0.001 compared with the control group. Statistical analysis in (B–D) was performed using an unpaired *t*‐test with Tukey's multiple comparisons test. G–J) ^^^ *p* < 0.05, ^^^^ *p* < 0.01, ^^^^^ *p* < 0.001 compared with the WT‐control group. ^*^ *p* < 0.05, ^**^ *p* < 0.01, ^***^ *p* < 0.001 compared with the WT‐LPS group. ^#^ *p* < 0.05, ^##^ *p* < 0.01, ^###^ *p* < 0.001 compared with the KO‐control group. All data in (G–J) were analyzed using one‐way ANOVA with Tukey's multiple comparisons test.

The results of multiplex IF staining demonstrated that microglia in the retina express PD‐L1 (**Figure** **5**A). Flow cytometry analysis further revealed a significant increase in PD‐L1 expression on retinal microglia/macrophages in CNV model mice (Figure 5B). Given the important role of microglia/macrophages in the RPE‐choroid during CNV progression,^[^ ^46^, ^62^, ^63^ ^]^ we also assessed PD‐L1 expression in these cells within the RPE‐choroid. Multiplex IF staining showed that microglia near the laser‐induced lesions in the RPE‐choroid express PD‐L1 (Figure 5C). Subsequently, we isolated microglia/macrophages from the RPE‐choroid using magnetic bead sorting and performed qPCR analysis. The qPCR results indicated a significant upregulation of PD‐L1 mRNA in RPE‐choroid microglia/macrophages following CNV induction (Figure 5D).

These findings demonstrate that both retinal and RPE‐choroid microglia/macrophages express PD‐L1 and that PD‐L1 expression is significantly increased under CNV pathological conditions. This suggests that the retinal PD‐L1 upregulation may be similar to previous study, which linked to response to inflammatory stimuli, transmitting negative regulatory signals that limit excessive activation of these cells, thus helping to control neuroinflammation.^[^ ^49^, ^61^ ^]^

Previous studies have demonstrated that PD‐L1 expressed on macrophages in vitro continuously transmits negative regulatory signals, thereby suppressing macrophage proliferation and the secretion of inflammatory cytokines. Blocking PD‐L1 or knocking out the PD‐L1 gene abolishes this regulatory mechanism, leading to heightened macrophage activation.^[^ ^64^, ^65^ ^]^ To further investigate whether PD‐L1 directly acts on microglia/macrophages, we conducted in vitro experiments using primary microglia derived from WT and PD‐L1 KO mice. The identity of primary microglia was confirmed by CD68 IF staining (Figure S4A, Supporting Information), and PD‐L1 expression was validated using Western blot analysis (Figure S4B, Supporting Information). LPS was used to simulate an inflammatory environment.

IF staining showed that PD‐L1 is expressed in WT primary microglia and is significantly upregulated following LPS stimulation (Figure 5E). This finding aligns with the observed upregulation of PD‐L1 in microglia/macrophages in the retinas and RPE‐choroid of CNV model mice in this study (Figure 5A–D). In contrast, primary microglia derived from PD‐L1 KO mice did not express PD‐L1 either before or after LPS stimulation (Figure 5F). These results were further corroborated by Western blot analysis (Figure S4C,D, Supporting Information).

To explore whether PD‐L1 directly regulates inflammatory responses, we conducted in vitro experiments using primary microglia from WT and PD‐L1 KO mice. PD‐L1 expression in WT microglia was significantly upregulated following LPS stimulation, consistent with ex vivo CNV findings. ELISA revealed that LPS‐induced secretion of proinflammatory cytokines (IL‐1β, IL‐6, TNF‐α) was further enhanced in PD‐L1 KO microglia, confirming the inhibitory role of PD‐L1. Interestingly, while activated microglia exhibited increased VEGF secretion, blocking PD‐L1 did not affect VEGF levels, indicating that PD‐L1 primarily modulates proinflammatory activity rather than proangiogenic functions. Additionally, exogenous PD‐1 significantly suppressed cytokine secretion in WT microglia but not in PD‐L1 KO cells, demonstrating that PD‐1 reinforces PD‐L1's inhibitory signals to mitigate inflammation.

These results demonstrate that disrupting PD‐L1 signaling significantly influences microglia and macrophages, causing increased activation and intensifying inflammatory responses. Furthermore, while activated microglia and macrophages secrete VEGF, this activity seems unrelated to PD‐L1 signaling. This suggests that the inflammatory and angiogenic roles of these cells are controlled by separate pathways,^[^ ^46^ ^]^ with PD‐L1 specifically regulating their inflammatory functions.

### 2.6 Microglia Depletion Abolishes the Effect of PD‐L1 on CNV in Mice

To investigate whether PD‐L1 regulates CNV in mice directly through microglia, we utilized a PLX5622 diet to deplete microglia.^[^ ^66^ ^]^ PLX5622 is an inhibitor specifically designed to target the colony‐stimulating factor‐1 receptor (CSF‐1R).^[^ ^67^, ^68^ ^]^ In WT mice, IF staining further confirmed that 1 week of PLX5622 treatment effectively depletes retinal microglia (Figure S5A,B, Supporting Information). qPCR analysis confirmed that 1 week of treatment with PLX5622 resulted in a significant downregulation of retinal microglia‐associated genes (Figure S5C–F, Supporting Information).

**Figure 6:**
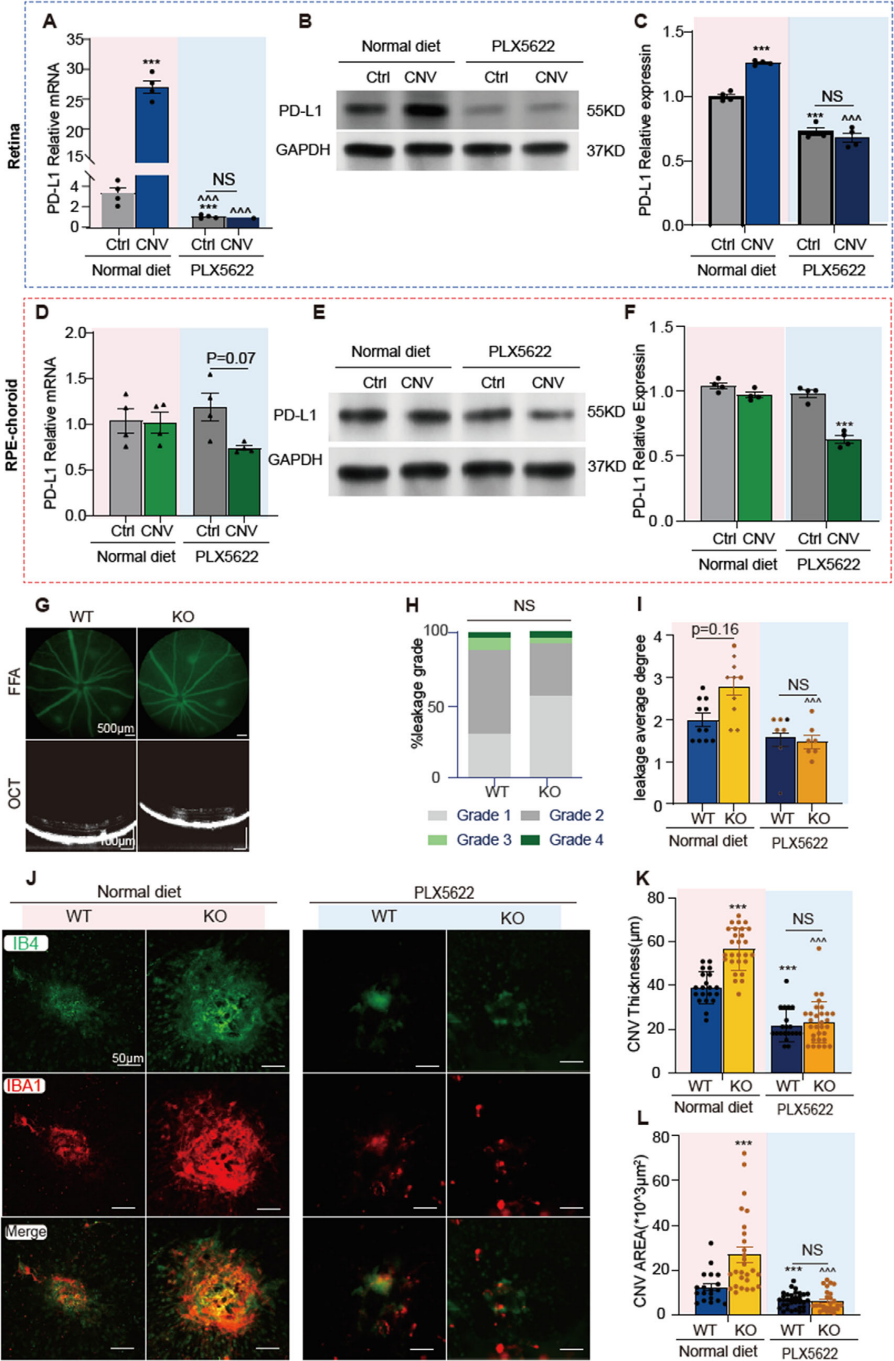
Microglia depletion abolishes the effect of PD‐L1 on choroidal neovascularization (CNV) in mice. A) Quantitative analysis of PD‐L1 mRNA expression in the retina of mice on two different diets, 3 days after laser injury. B) Representative Western blot images showing retinal PD‐L1 protein levels in mice on a normal diet or PLX5622 diet, 3 days post‐laser injury. C) Quantitative analysis of retinal PD‐L1 protein levels in mice fed a normal diet or PLX5622 diet. D) PD‐L1 mRNA expression in retinal pigment epithelium (RPE) choroid tissues, analyzed using the same method as in panel (A). E) Representative Western blot images showing PD‐L1 protein levels in the RPE‐choroid of mice on two different diets, 3 days after laser injury. F) Quantitative analysis of PD‐L1 protein levels in RPE‐choroid tissues, analyzed using the same method as in panel C (*n* = 4 mice per group). G) Top panels show representative fundus fluorescein angiography (FFA) images from wild‐type (WT) and PD‐L1 knockout (KO) mice fed with PLX5622 diet at 7 days post‐laser injury. Bottom panels show the example images of optical coherence tomography (OCT) scan. H) Summaries of the FFA grade scores of each laser spot from WT and PD‐L1 KO mice fed with PLX5622 diet at 7 days post‐laser injury (*n* = 23 and 25 laser spots from WT and KO mice, respectively). I) Summaries of the average leakage grade from WT and PD‐L1 KO mice fed with normal or PLX5622 diet at 7 days post‐laser injury. For normal diet group, *n* = 11 WT mice and 10 KO mice. For PLX5622 diet group, *n* = 6 WT mice and 6 KO mice. J) Immunofluorescence (IF) staining of IB4 and IBA1 in RPE/choroidal flat mounts from laser‐induced CNV mice. The left two panels display RPE/choroidal images of WT and PD‐L1 KO mice from the normal diet group, and the right two panels exhibit RPE/choroidal images of WT and PD‐L1 KO mice from the PLX5622 diet group. K,L) Quantifications of CNV thickness and area in RPE/choroidal flat mounts at 7 days after laser injury. For normal diet, *n* = 7 WT mice and 10 KO mice. For PLX5622 diet group, *n* = 6 WT mice and 6 KO mice. Data are presented as mean ± SEM. Chi‐square test for FFA grade scores in (H). Nonparametric test for leakage average degree in (I). One‐way ANOVA with Tukey's multiple comparisons test was used for analyzing PD‐L1 expression levels in (A,C,D,F), as well as CNV thickness in (K) and CNV area in (L). ^*^ *p* < 0.05; ^**^ *p* < 0.01; and ^***^ *p* < 0.001 compared to the normal food WT group. ^^^ *p* < 0.05; ^^^^ *p* < 0.01; and ^^^^^ *p* < 0.001, compared to the normal food KO group. Source data are provided as a Source Data file.

After depleting microglia using PLX5622, PD‐L1 mRNA and protein levels in the retina were significantly reduced compared to those in mice on a normal diet (including WT‐control and WT‐CNV groups) and did not increase notably following laser‐induced CNV (**Figure** **6**A–C). In the RPE‐choroid, PD‐L1 mRNA and protein levels in PLX5622‐control mice showed no significant differences compared to normal diet groups. However, in the PLX5622‐CNV group, PD‐L1 expression in the RPE‐choroid exhibited a decreasing trend, with PD‐L1 protein levels in the choroid significantly lower than those in PLX5622‐control mice (Figure 6D–F). These findings suggest that PD‐L1 in the retina is predominantly expressed by microglia/macrophages, while in the RPE‐choroid, its expression is only partially attributed to these cells.

To further investigate the effects of microglial depletion on inflammation, we assessed inflammatory markers in the retina and RPE‐choroid of WT and PD‐L1 KO mice. The mRNA levels of IL‐1β, IL‐6, and TNF‐α were significantly reduced in PLX5622‐treated mice compared to those on a normal diet, with no significant differences between WT and PD‐L1 KO mice (Figure S5G,H, Supporting Information).

Consistent with these findings, FFA analysis revealed that after PLX5622 treatment, only ≈4% of WT and PD‐L1 KO mice exhibited grade 4 leakage^[^ ^53^ ^]^ (Figure 6G,H), compared to 9.5% and 29.7%, respectively, in normal diet groups (Figure 2A,B). Furthermore, the average leakage grades of CNV were significantly reduced in both WT and PD‐L1 KO mice (Figure 6G,I). OCT analysis confirmed a significant reduction in CNV thickness in PLX5622‐treated WT and KO mice compared to normal diet groups (Figure 6G,K). IB4 staining of RPE‐choroid flat mounts further demonstrated a marked decrease in CNV lesion areas in PLX5622‐treated mice (Figure 6J,L). Importantly, no significant differences in CNV leakage or lesion size were observed between WT and PD‐L1 KO mice after PLX5622 treatment (Figure 6H,I,K,L).

These results indicate that microglia/macrophage depletion via PLX5622 significantly reduces inflammation in the retina and RPE‐choroid, suppressing CNV formation and leakage. Moreover, microglial/macrophage depletion reverses the increased inflammation and exacerbated CNV observed with PD‐L1 blockade.

Collectively, these findings suggest that the regulatory effects of PD‐L1 on CNV in mice are mediated through microglia/macrophages.

### 2.7 PD‐L1 Modulates CNV Progression by Regulating the MAPK/ERK Pathway in Microglia

**Figure 7:**
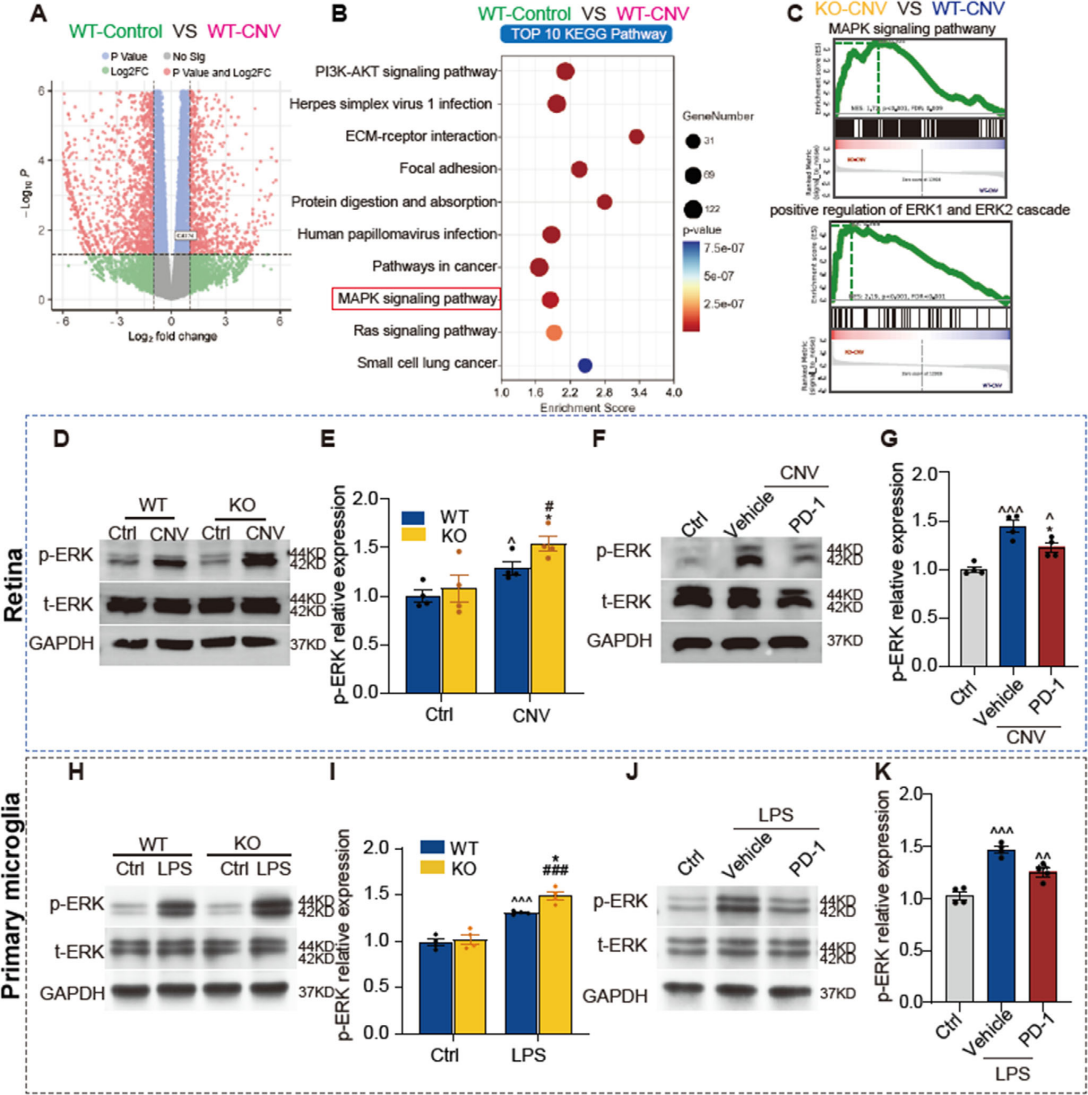
PD‐L1 modulates choroidal neovascularization (CNV) progression by regulating the MAPK/ERK pathway in microglia. A) Volcano plot obtained from DESeq2 analysis of wild‐type (WT) mice retina at 3 days after laser injury compared with WT mice without laser intervention. B) KEGG pathway analysis of the differential expression genes (DEGs). C) Gene set enrichment analysis (GSEA) pathway analysis of the DEGs from PD‐L1 knockout (KO) mice retina and WT mice retina at 3 days after laser injury. D) Western blot images and E) statistical results of the expression levels of p‐ERK in the retinas of control mice and mice with 3 days after laser treatment, for both WT and PD‐L1 KO mice (*n* = 4 mice per group). F) Western blot images and G) statistical result of the p‐ERK expression in the retinas from WT mice of the control, CNV + vehicle, CNV + PD‐1 groups at 3 days after laser injury (*n* = 4 mice per group). H–K) Same as (D–G), but for primary microglia. Data are presented as mean ± SEM. One‐way ANOVA with Tukey's multiple comparisons test was used for statistical analyses, ^^^ *p* < 0.05, ^^^^ *p* < 0.01, and ^^^^^ *p* < 0.001, compared to the WT‐control group. ^*^ *p* < 0.05, ^**^ *p* < 0.01, and ^***^ *p* < 0.001 compared to the WT‐CNV or WT‐LPS group. ^#^ *p* < 0.05, ^##^ *p* < 0.01, and ^###^ *p* < 0.001 compared to the KO‐control group. Source data are provided as a Source Data file.

To elucidate the specific molecular mechanisms underlying the laser‐induced CNV and investigate the influence of PD‐L1 on retinal microglial cells in the CNV model, we conducted RNA‐seq analysis on retinal tissues. Firstly, we compared the differential gene expression profiles between two distinct groups: WT mice at 3 days post‐laser injury (WT‐CNV) and control mice without laser intervention (WT‐Control) (**Figure** **7**A). PD‐L1 mRNA (coded by *Cd274* gene) was upregulated in the WT‐CNV group (Figure 7A), consistent with our previous findings from WB and qPCR (Figure 1A,B). KEGG pathway analyses unveiled a substantial enrichment of differentially expressed genes, primarily associated with inflammation and neovascularization (Figure 7B). We conducted a comprehensive pathway enrichment analysis using gene set enrichment analysis (GSEA) between WT (WT‐CNV) and KO (KO‐CNV) mice at 3 days after laser injury. Notably, our analysis revealed a significant upregulation of MAPK/ERK pathway in the retinas of KO mice compared to WT mice (Figure 7C).

The MAPK signaling pathway comprises a network of critical components including p38, extracellular signal‐regulated kinases (ERK), and c‐Jun N‐terminal kinase (JNK).^[^ ^69^, ^70^ ^]^ Activation of MAPK pathway assumes a central role in regulating inflammatory responses and innate immunity.^[^ ^69^, ^71^ ^]^ Previous studies showed that inhibiting PD‐L1 leads to an upregulation of phosphorylated ERK^[^ ^72^, ^73^ ^]^ and influences the functions of microglial cells.^[^ ^43^ ^]^ Based on these studies and our RNA‐seq analysis, we inferred that PD‐L1 exerts an influence on CNV via the ERK pathway‐based microglial activation. We employed WB to evaluate the p‐ERK level in the retinas following laser‐induced injury and observed a significant upregulation of retinal p‐ERK level in both WT and PD‐L1 KO mice following laser photocoagulation (Figure 7D,E). Notably, KO mice had a higher elevation of p‐ERK in comparison to WT mice (Figure 7D,E).

Previous results demonstrated that PD‐1 suppresses microglial activation and inflammation via PD‐L1. To investigate whether PD‐1 exerts its effects through the downstream ERK pathway after binding to PD‐L1, Western blot analysis was performed. In the laser‐induced CNV model, PD‐1 significantly reduced ERK phosphorylation in the retina (Figure 7F,G), consistent with the observation that PD‐L1 KO further increased p‐ERK levels in the retina of CNV model mice (Figure 7D,E).

In retinal homogenates from CNV‐induced PD‐L1 KO mice, we observed further activation of the p‐ERK pathway. Given the diverse cell types present in the retina, we designed in vitro experiments to specifically assess the effect of PD‐L1 on the ERK pathway in microglia. Consistent with previous studies,^[^ ^74^ ^]^ LPS stimulation significantly increased p‐ERK protein levels in primary microglia (isolated from WT and PD‐L1 KO mice) (Figure 7H,I). Furthermore, in line with our in vivo findings, LPS induced a more pronounced increase in p‐ERK expression when PD‐L1 signaling was blocked (Figure 7H,I). To determine whether PD‐1 directly modulates the ERK signaling pathway in microglia, primary microglia derived from WT and PD‐L1 KO mice were pretreated with PD‐1 protein or control vehicle 2 h before LPS stimulation. PD‐1 significantly inhibited LPS‐induced activation of the ERK pathway in WT primary microglia (Figure 7J,K).

In conclusion, our findings demonstrate that the MAPK/ERK pathway is activated in both the retinas of CNV mice and microglia following inflammatory stimulation. Reactively upregulated PD‐L1 provides sustained negative regulatory signals that partially suppress ERK activation. Disruption of PD‐L1 signaling removes this negative feedback, exacerbating neuroinflammation mediated by microglia and macrophages. Additionally, activation of PD‐L1 by PD‐1 further attenuates MAPK/ERK pathway activation in the retinas of CNV mice and in microglia.

### 2.8 ERK Inhibitor Suppresses Microglial Activation and Reduces CNV Lesion Area

**Figure 8:**
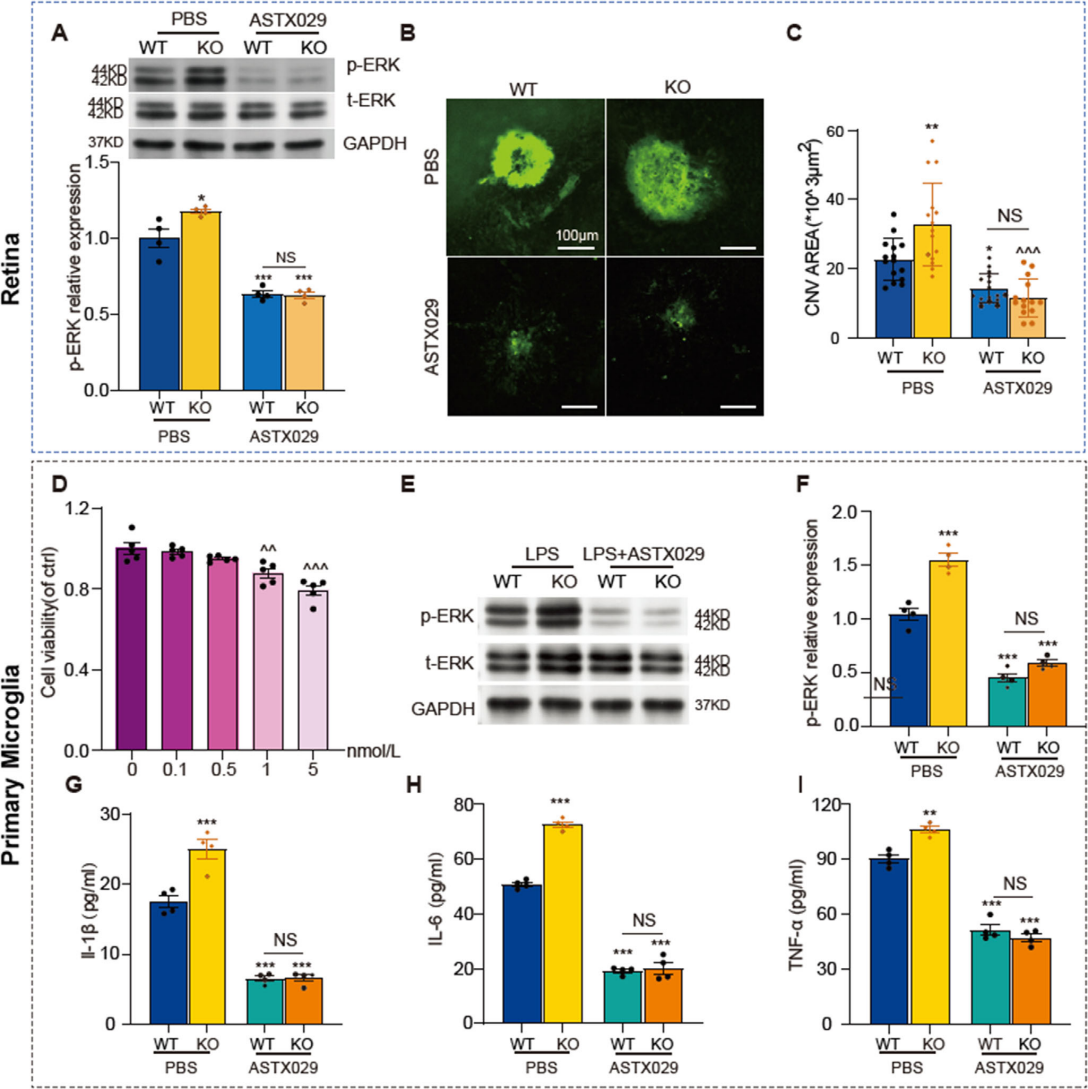
ERK inhibitor suppresses microglial activation and reduces choroidal neovascularization (CNV) lesion area. A) Representative Western blot images (top) and statistical analysis (bottom) of p‐ERK expression in the retinas of wild‐type (WT) and PD‐L1 knockout (KO) mice, 3 days post‐laser treatment, following intravitreal injection of ASTX029 or PBS. B) IB4 staining of retinal pigment epithelium (RPE)/choroidal flat mounts from WT and KO mice, 7 days after laser injury, following intravitreal injection of ASTX029 or PBS (*n* = 4 mice per group). C) Quantification of the laser‐induced CNV area. D) Effects of different concentrations of ASTX029 on primary microglia derived from WT mice. E) Representative Western blot images and (F) statistical analysis of p‐ERK expression levels. G–I) Corresponding analyses of IL‐1β, IL‐6, and TNF‐α mRNA expression in LPS‐stimulated primary microglia samples derived from WT or KO mice. Data are presented as mean ± SEM (*n* = 4 mice or four samples of primary microglia per group). Statistical significance is denoted as follows: ^^^ *p* < 0.05, ^^^^ *p* < 0.01, ^^^^^ *p* < 0.001 (compared to the WT‐control group); ^*^ *p* < 0.05, ^**^ *p* < 0.01, ^***^ *p* < 0.001 (compared to the WT‐CNV or WT‐LPS group); ^#^ *p* < 0.05, ^##^ *p* < 0.01, ^###^ *p* < 0.001 (compared to the KO‐control group). Statistical analyses were performed using one‐way ANOVA followed by Tukey's multiple comparisons test.

To investigate whether PD‐L1 specifically regulates microglial activation through the MAPK pathway, we employed the ERK pathway inhibitor ASTX029.^[^ ^75^ ^]^ ASTX029 was intravitreally injected into WT and PD‐L1 KO mice immediately following CNV induction. On the third day post‐laser injury, Western blot analysis revealed a significant reduction in retinal p‐ERK levels in both WT and KO mice treated with ASTX029 (**Figure** **8**A). IB4 staining of RPE‐choroid flat mounts demonstrated a substantial decrease in CNV lesion areas in ASTX029‐treated mice compared to PBS‐treated controls (Figure 8B,C). Furthermore, the differences in retinal p‐ERK levels and CNV lesion sizes between WT and KO mice were abolished by ASTX029 treatment (Figure 8B,C, Supporting Information).

To further confirm whether ERK pathway inhibition directly suppresses microglial secretion of inflammatory cytokines, we treated primary microglia derived from WT mice with ASTX029 in vitro. CCK‐8 assays indicated that 0.5 nmol L^−1^ of ASTX029 had no significant impact on microglial viability, and this concentration was used for subsequent experiments (Figure 8D). Western blot analysis showed that ASTX029 significantly reduced p‐ERK levels in LPS‐stimulated primary microglia and suppressed the secretion of IL‐1β, IL‐6, and TNF‐α (Figure 8E–I). Notably, ASTX029 also reversed the differences in ERK activation between microglia derived from WT and KO mice, consistent with the ex vivo findings (Figure 8A).

These results collectively suggest that PD‐L1 regulates microglia‐mediated neuroinflammation through the MAPK pathway, highlighting its role as a key modulator of the MAPK/ERK signaling cascade.

## 3 Discussion

Neuroinflammation and dysregulation of neuroimmune processes are prominent features in various retinal degenerative disorders, including AMD.^[^ ^20^ ^]^ ICPs have emerged as crucial regulators of inflammation and immune responses, exhibiting significant therapeutic potential in diverse fields, such as oncology^[^ ^76^ ^]^ and inflammatory disorders.^[^ ^77^ ^]^ Among these, the PD‐1/PD‐L1 axis stands out as a pivotal molecular component.^[^ ^38^ ^]^ This study investigated the role and possible mechanisms of PD‐L1 in the laser‐induced CNV model. We observed that retinal PD‐L1 expression, particularly on microglia/macrophages, was increased following the laser‐induced CNV. Blockage of PD‐L1 by PD‐L1 genetic ablation or antibody exacerbated the pathological neovascularization in the CNV model, which may due to retinal microglia/macrophages activation and the elevation of neuroinflammation via ERK‐related pathway. In addition, intravitreal delivery of PD‐1, the ligand of PD‐L1, reduced retinal p‐ERK activation and neuroinflammation to mitigate vascular leakage and neovascularization in the CNV model. These evidences highlight the pivotal role of ICPs in regulating innate neuroimmune responses and neuroinflammatory processes in the laser‐induced CNV mouse model, and suggest that targeting the PD‐L1 pathway may be a potent strategy to treat the NVAMD.

In the central nervous system, PD‐L1 exhibits a typical upregulation in response to inflammatory stimuli, which will reduce neuroinflammation and mitigate disease severity.^[^ ^34^ ^]^ Previous investigations have substantiated an augmented expression of PD‐L1 in inflamed retinas,^[^ ^78^ ^]^ although retinal PD‐L1 level was reported to be diminished in the proliferative diabetic retinopathy.^[^ ^79^ ^]^ Our retinal RNA‐seq, PCR, and WB analyses disclosed a noteworthy elevation of retinal PD‐L1 subsequent to the laser‐induced CNV (Figures 1A,B, and 7A).

PD‐L1 is expressed across multiple cell types within CNV lesions.^[^ ^80^ ^]^ In conditions such as postoperative brain injury^[^ ^61^ ^]^ and spinal cord injury,^[^ ^43^ ^]^ PD‐L1 on microglia can suppress excessive activation and mitigate neuroinflammation. Consistent with these findings, this study demonstrates that PD‐L1 is expressed on microglia/macrophages in the retina and RPE‐choroid of mice, with expression levels increasing following CNV induction (Figure 5A–D). Additionally, primary microglia from WT mice exhibited PD‐L1 expression, which was enhanced post‐inflammatory stimulation (Figure 5E, Figure S4C,D, Supporting Information). Blocking PD‐L1 promoted the secretion of inflammatory cytokines by microglia/macrophages (Figure 5G–I), whereas PD‐1 activation of PD‐L1 inhibited these cells and reduced cytokine release (Figure 5G–I). Furthermore, the overall retinal PD‐L1 expression patterns mirrored those observed in microglia/macrophages, with significant upregulation following either CNV induction or inflammatory stimuli. Removal of microglia/macrophages led to a marked decrease in retinal PD‐L1 levels, indicating that PD‐L1 expression in the retina predominantly originates from these cells.

Despite an increase in PD‐L1 expression among activated microglia/macrophages within the RPE‐choroid in CNV model mice (Figure 5C,D), overall PD‐L1 levels in the RPE‐choroid tissue did not significantly alter (Figure 1C,D). After depleting microglia/macrophages, a downward trend in PD‐L1 levels in RPE‐choroid tissue was observed (Figure 6D–F). This discrepancy may stem from other cell types within the RPE‐choroid, such as RPE cells, which are also capable of expressing PD‐L1.^[^ ^44^, ^81^ ^]^ RPE damage constitutes a pivotal factor in the pathogenesis of NVAMD^[^ ^82^ ^]^ potentially leading to inconsistent PD‐L1 expression patterns between microglia/macrophages and the overall tissue. Future research should delve deeper into PD‐L1 expression across various cell types within the RPE‐choroid and specifically quantify PD‐L1 in RPE‐choroid microglia/macrophages to elucidate the role of PD‐L1 in modulating inflammation in the CNV model more precisely.

During activation, microglia/macrophages release a significant amount of inflammatory cytokines, major contributors to tissue damage and vascular leakage.^[^ ^46^ ^]^ Reactively upregulated PD‐L1 can inhibit the activation levels and cytokine secretion of microglia/macrophages.^[^ ^61^, ^83^ ^]^ In alignment with prior research, our study shows that blocking PD‐L1 signaling leads to enhanced activation of microglia/macrophages in the retina and RPE‐choroid of CNV mice, and consequently higher levels of inflammation in these tissues (Figure 4). Further, in vitro studies illustrate that inhibiting PD‐L1 signaling results in increased secretion of proinflammatory cytokines from microglia following LPS stimulation (Figure 5G–I), whereas activating PD‐L1 markedly reduces cytokine output (Figure 5G–I). These observations suggest that PD‐L1's protective mechanism in CNV likely involves suppressing inflammatory cytokine secretion by microglia/macrophages, thereby mitigating inflammation‐associated vascular leakage and pathological angiogenesis.

Moreover, to assess whether PD‐L1's protective effects specifically rely on microglia/macrophages, we utilized PLX5622 to deplete these cells in mice. Following microglial/macrophage depletion, we observed decreased levels of inflammatory cytokines, reduced vascular leakage, and a diminished extent of CNV (Figure 6G–L, Figure S5G,H, Supporting Information), reinforcing that moderating inflammation in these tissues is an effective strategy for treating CNV. Additionally, once microglia/macrophages were depleted, the differences in local inflammation, vascular leakage, and neovascular area between PD‐L1 KO and WT mice were eliminated, indicating that PD‐L1's role in modulating CNV predominantly involves these cells.

Despite the predominant use of anti‐VEGF therapy in the management of CNV,^[^ ^84^ ^]^ activated microglia/macrophages not only produce increased inflammatory cytokines but also secrete VEGF.^[^ ^85^ ^]^ According to Anne Wolf et al., the proinflammatory and VEGF‐secreting functions of microglia may be regulated through distinct mechanisms.^[^ ^46^ ^]^ Our findings confirm that PD‐L1 signaling specifically inhibits cytokine production without affecting VEGF levels in vitro in microglia and in the retina of CNV model mice (Figure 5J, Figure S3, Supporting Information), indicating that PD‐L1's inhibitory effect on CNV does not operate via anti‐VEGF pathways. Given the efficacy of anti‐VEGF in suppressing CNV, future clinical strategies might explore the potential synergistic effects of combining PD‐L1 and anti‐VEGF therapies, potentially mirroring dual‐specificity antibody treatments used in cancer therapy.^[^ ^86^ ^]^ Additionally, the cellular distribution of VEGF signaling warrants further exploration to fully understand its role in the CNV.

It is important to note that extensive research has demonstrated that CNV in mice often presents with significant local inflammation immediately after model induction, with microglia/macrophage activation peaking on the third day post‐laser treatment and CNV formation peaking on the seventh day.^[^ ^52^ ^]^ This suggests that the inflammatory processes in CNV may precede and continually influence pathological neovascularization. Our results indicate that immediate post‐laser PD‐1 injection can suppress CNV by inhibiting inflammation, highlighting the potential of early anti‐inflammatory treatments to improve clinical outcomes.

Through RNA sequencing, we observed that laser‐induced CNV significantly influenced the MAPK pathway, a common target for mitigating neuroinflammation (Figure 7A–C). The ERK subgroup, a crucial component of the MAPK family, holds particular significance. Activation of the ERK pathway has also been observed in retinal degenerative diseases, including AMD, and inhibiting ERK has been shown to alleviate retinal neuroinflammation.^[^ ^87^ ^,154]^ Consistent with previous studies, our research revealed a significant upregulation of the ERK pathway in the retinas of CNV mice and in microglia activated by LPS. Inhibition of the ERK pathway effectively attenuates CNV and reduces proinflammatory cytokine secretion by microglia (Figure 8). These findings suggest that activation of the ERK pathway plays a crucial role in the activation of microglia/macrophages and the inflammatory response in CNV, making it a potential therapeutic target.

The relationship between PD‐L1 and the ERK pathway is noteworthy. Previous studies have documented that PD‐L1 activation in spinal cord injury can inhibit the MAPK pathway, specifically ERK.^[^ ^43^ ^]^ However, additional research has demonstrated that the PD‐1 active PD‐L1 signaling, leading to the increased phosphorylation of ERK in T cells.^[^ ^88^ ^]^ These suggest that the signaling mechanisms of PD‐L1 may vary across diverse tissues and diseases. (Figure 7C–E). In our study, we found that blocking PD‐L1 with antibodies or knocking out the PD‐L1 gene further enhanced the activation of the MAPK/ERK pathway in the retinas of CNV mice and in LPS‐stimulated microglia (Figure 7D,E,H,I). Conversely, intervention with PD‐1 resulted in a decrease in ERK phosphorylation levels (Figure 7F,G,J,K). These findings align with those of Hartley et al,^[^ ^89^ ^]^ suggesting that PD‐L1, as a negative co‐stimulatory ICP molecule, can continuously suppress the activation of certain intracellular signaling pathways, thus regulating cellular functions. In our experiments, we observed that the upregulated PD‐L1 could only partially limit ERK pathway activation, and even enhanced PD‐L1 signaling via PD‐1 could not fully suppress ERK activation. This may be due to the complex regulatory mechanisms of the ERK pathway,^[^ ^90^ ^]^ which prevent PD‐L1 signaling from completely reversing ERK pathway activation in proinflammatory microglia.

This study is subject to several limitations. NVAMD is a multifaceted condition influenced by a variety of factors, including age, metabolism, genetics, and environmental elements.^[^ ^3^, ^13^ ^]^ However, our employed model primarily replicates one crucial facet of NVAMD observed in humans, the development of CNV, which is induced through laser‐induced injury. This model initiates a wound‐healing response subsequent to an insult at the level of Bruch's membrane, heavily relying on an inflammatory process.^[^ ^42^ ^]^ Consequently, it underscores the predominant role of ICP regulation. When contemplating potential therapeutic targets, it is crucial to consider additional variables such as genetics and age. In addition, due to the expression of PD‐L1 in various cell types,^[^ ^33^, ^43^, ^44^ ^]^ additional experiments employing retinal microglia conditional KO mice are imperative to ascertain the precise role of PD‐L1 in CNV. In our experiments, we used global PD‐L1 KO mice to demonstrate that PD‐L1 deficiency exacerbates laser CNV injury and angiogenesis. However, this global KO approach does not elucidate the specific role of microglial PD‐L1. Further experiments employing conditional KO models, such as microglia‐specific PD‐L1 KO mice, are needed to convincingly establish the microglial‐specific effects of PD‐L1 on angiogenesis.

In summary, our research provides compelling evidence for the involvement of ICPs in the regulation of CNV. We propose that PD‐L1 serves as a regulatory node, modulating retinal microglia functions via ERK. Specifically, PD‐1, through the PD‐L1/ERK signaling pathway, mitigates retinal neuroinflammation, thus providing an effective method for suppressing CNV. The manipulation of ICPs presents a promising and indispensable role in the NVAMD treatment.

## 4 Experimental Section

### Animals

The experiment utilized adult mice aged 8–10 weeks. PD‐L1 KO mice with a C57BL/6 background were obtained from Cyagen Biosciences Inc. (Suzhou, China), while PD‐L1 WT mice with a genetically equivalent background served as the control group. To minimize potential experimental biases resulting from gender differences, an equal number of male and female mice were employed unless otherwise specified. Mice were housed in a controlled environment at a temperature of 22 ± 2 °C with a 12:12‐h light–dark cycle, and they had free access to ample provisions of food and water. Genotyping was performed using PCR analysis on tail DNA samples.^[^ ^91^ ^]^ All experimental procedures adhered to the guidelines established by the Animal Care and Use Committee of Fudan University (Shanghai, China; approval No. 2023DW008).

### Laser Photocoagulation

Mice were anesthetized by intraperitoneal injection of 1% pentobarbital sodium (40 mg kg^−1^). Pupil dilation was achieved using 0.5% tropicamide eye drops. The laser photocoagulation procedure followed the guidelines described in reference.^[^ ^42^ ^]^ A diode laser system, designed in a slit‐lamp style (Quantel Medical Vitra), employing a green laser at 532 nm with a power of 100 mW, a duration of 100 ms, and a spot size of 100 µm, was utilized. To facilitate fundus observation, a glass coverslip was positioned in front of each eye. Four laser spots were administered around the optic nerve. Successful laser photocoagulation was determined by the minimal presence of bubbles preceding the laser spot, indicating the rupture of Bruch's membrane.^[^ ^42^ ^]^

### Intravitreal Delivery

For intravitreal delivery, mice were anesthetized, and their pupils were dilated using the aforementioned methods. A 34‐gauge needle was inserted into the vitreous space approximately 1.5 mm below the limbus, and 1 µL solution was administered bilaterally with a Nanofil syringe.^[^ ^92^ ^]^ The following groups were designated: anti‐PD‐L1 (5 ng PD‐L1 antibody, clone 10F.9G2, Biolegend), PD‐1 (5 ng PD‐1 protein, RP01170, Abclone), and vehicle (saline only), ASTX029 (50 µm, S20663, MedMol.).^[^ ^93^ ^]^

### Fundus Photography and FFA

The fundus photography and FFA examination were conducted at 7 days post‐laser photocoagulation.^[^ ^46^ ^]^ Mice were appropriately immobilized and subjected to anesthesia. Pupil dilation was achieved using a combination of tropicamide eye drops. Mice were positioned correctly, carbachol eye drops were applied to the cornea. Subsequently, the cornea was brought into contact with the microscope lens of the Optoprobe Science LTD small animal fundus imaging system (OPTO‐RIS). The experimental table and lens focus were adjusted according to facilitate optimal imaging conditions, following which photographs were taken.

We performed FFA to evaluate vascular leakage at 7 days after laser treatment.^[^ ^46^ ^]^ After anesthesia and pupil dilation, the mice received an intraperitoneal injection of 1.7 mL kg^−1^ of a 2% sodium fluorescein solution. Subsequent photographs were taken to document the FFA images. Both eyes were photographed to ensure comprehensive data collection. Following the experiment, eyes were rinsed with saline solution, and levofloxacin eye drops were administered as a preventive measure against potential infections. Retinal arterial filling represents the early phase of angiography ((1–2 min after fluorescein injection)), whereas complete filling of both retinal arteries and veins marks the late phase of angiography (4–5 min after fluorescein injection).^[^ ^94^, ^95^ ^]^

The assessment of fluorescein leakage intensity in the study followed the established Takehana grading criteria.^[^ ^42^ ^]^ The criteria provided a systematic framework for classifying the observed patterns: Grade 1, denoted as “no leakage,” described the presence of faint high fluorescence or speckled fluorescence; Grade 2, referred to as “questionable leakage,” indicated the absence of size or intensity increase in late‐phase high fluorescence; Grade 3, termed “leaky,” described the situation where the fluorescence intensity within the high fluorescence area increased while the size remained unchanged; Grade 4, known as “pathologically significant leakage,” characterized both an increase in fluorescence intensity and size. The average grade represents the mean value of leakage grades for each laser spot on the retina. Adhering to this standardized classification scheme allowed for a more precise and consistent evaluation of fluorescein leakage intensity in the experimental analysis.

### Optical Coherence Tomography (OCT)

We employed OCT to quantify the CNV thickness and detect the presence of sub‐retinal fluid.^[^ ^96^ ^]^ After administering anesthesia and pupil dilation, surface anesthesia was further achieved using proparacaine hydrochloride eye drops. Subsequently, mice were positioned on an elevated table. The eye's position was carefully adjusted, and medical carbomer eye drop gel was applied to the cornea under examination. The Ultramicro Ophthalmol Imaging System (ISOCT, OPTOPROBE) light source was finely tuned to focus, and the lens was adjusted to focus on the retina. OCT of the retina was then performed. Both eyes of each animal were photographed for comprehensive data collection. CNV thickness was measured by manually drawing line segments perpendicular to the RPE at the site of CNV in OCT images.

### Flat Mounts, Immunohistochemistry, and Image Analysis

Mice were euthanized using cervical dislocation, and their eyeballs were removed and fixed in 4% paraformaldehyde (PFA) for 2 h. The retina and RPE‐choroid complex were isolated and subjected to permeabilization and blocking. Following this, the primary antibody, anti‐iba1 (Wako, 019–19741), was applied to the samples and allowed to incubate for 24 h at 4 °C. Subsequent to the primary antibody incubation, the samples were subsequently incubated with a secondary antibody, goat anti‐rabbit AlexaFluorTM 647 (A21244, Invitrogen), for 2 h. In the case of RPE/choroid samples, staining was conducted using IB4 (Sigma‐Aldrich). Following three PBS washes, the retina and RPE/choroid samples were carefully positioned flat on glass microscope slides for imaging purposes. The area of laser lesions was quantitatively evaluated using ImageJ in a blinded manner.

### Flow Cytometry Staining and Analysis

Tissues were rinsed in 1% three‐antibody PBS buffer, followed by fragmentation using ophthalmic scissors. After digestion with trypsin and collagenase, the tissue fragments were washed and resuspended in high‐glucose DMEM culture medium with FBS. The resulting cell suspension was filtered and prepared for analysis. Cells were incubated with specific antibodies, followed by centrifugation and resuspension in flow cytometry staining buffer. Flow cytometry analysis was performed to measure the expression levels of CD11B (ab8878, abcam) and PD‐L1 (ab205921, abcam).

### Transcription Analysis by RT‐qPCR

Tissue samples obtained from ex vivo mouse experiments were promptly collected and immediately snap‐frozen. Total RNA extraction was carried out using the Tissue RNA Purification Kit from EZBioscience (USA). Subsequently, cDNA was synthesized using the EZscript Reverse Transcription Mix II with gDNA Remover (EZBioscience). Gene expression analysis was conducted employing the Bio‐Rad RT‐PCR System (Hercules, USA) with 2× SYBR Green qPCR Master Mix. β‐actin served as the internal control for normalization purposes. The relative changes in gene expression were quantified using the 2^−ΔΔCt method. Each sample was assayed in triplicate, and the presented data represents the mean of three independent assessments. Primer information is provided in Table S1 (Supporting Information).

### Western Blot Analysis

Fresh retinal/RPE‐choroid tissue was homogenized using a lysis buffer (P0013B, Beyotime) for protein extraction. The protein concentration of each sample was determined with a BCA protein assay kit (P0012S, Beyotime). Equal amounts of protein were loaded onto 10% SDS‐PAGE gels and subsequently transferred to PVDF membranes. After blocking with 5% milk for 2 h at room temperature, the membranes were incubated overnight at 4 °C with primary antibodies. Following this, they were incubated with horseradish peroxidase (HRP) conjugated secondary antibodies (A0216 or A0208, Beyotime) for 2 h at room temperature. The primary antibodies used in Western blotting included: ERK1/ERK2 Rabbit pAb (ABclone, A16686); Phospho‐ERK1‐T202/Y204 + ERK2‐T185/Y187 Rabbit mAb (ABclone, AP0974); Anti‐PD‐L1 antibody (abcam, ab213480); and mouse anti‐GAPDH (60004‐1‐Ig, Proteintech). Immunoblots were visualized using an ECL Kit Chemiluminescence (P0018S, Beyotime), and Image‐J software 1.80 (National Institutes of Health, USA) was employed for data analysis.

### 3D Image Reconstruction and Analysis

Z‐stack confocal images were obtained using either a Zeiss LSM 900 confocal microscope with a Plan‐Apochromat ×20/1.4 NA objective. Three‐dimensional retinal images were constructed using the Surface module within Imaris 9.5.0 software (Bitplane, Switzerland).

### RNA Sequencing (RNA‑seq)

Total RNA was extracted using the TRIzol reagent (Invitrogen, CA, USA) following the manufacturer's protocol. RNA purity and quantification were assessed using the NanoDrop 2000 spectrophotometer (Thermo Scientific, USA). RNA integrity was determined using the Agilent 2100 Bioanalyzer (Agilent Technologies, Santa Clara, CA, USA). Subsequently, libraries were prepared using the VAHTS Universal V6 RNA‐seq Library Prep Kit in accordance with the manufacturer's instructions. Transcriptome sequencing and analysis were conducted by OE Biotech Co., Ltd. (Shanghai, China). The libraries were sequenced on an Illumina NovaSeq 6000 platform, generating 150 bp paired‐end reads. Differential expression analysis was performed using DESeq2.^[^ ^97^ ^]^ A threshold for significantly DEGs was set at Q value < 0.05 and fold change > 2 or fold change < 0.5. A heatmap was generated using an online platform for data analysis and visualization available at https://www.bioinformatics.com.cn (last accessed on 10 July 2023). For further analysis, GO enrichment analysis^[^ ^98^ ^]^ and KEGG pathway enrichment analysis^[^ ^99^ ^]^ of DEGs were conducted to identify significantly enriched terms based on the hypergeometric distribution. GSEA was carried out using GSEA software.^[^ ^100^ ^]^

### Primary Microglia Isolated and Culture

Primary microglia were isolated from 1–10‐day‐old neonatal mice (C57BL/6J WT or PD‐L1 KO). The mice were disinfected with 75% ethanol for 5–10 min, then transferred to pre‐chilled PBS for cerebral cortex dissection. Cortical tissue was washed three times with PBS, minced into ≈1 × 1 mm pieces, and digested in 0.25% trypsin with collagenase at 37 °C in a shaking water bath for 30 min. Digestion was terminated by adding FBS, and the tissue was gently triturated to form a single‐cell suspension. The suspension was filtered through a 100 µm strainer, centrifuged at 1000 rpm for 5 min, and the cell pellet was resuspended in DMEM/F12 medium supplemented with 10% FBS and 50 ng mL^−1^ recombinant mouse CSF‐1. The cells were plated in T25 flasks, evenly distributed by gentle shaking, and cultured at 37 °C in a 5% CO₂ incubator. After 24 h, the medium was replaced, and cell growth was monitored. Half‐medium changes were performed every 2 days. By days 7–9, cells exhibited stratified growth, and at day 14, flasks were placed on a shaker at 220 rpm for 2–4 h to detach microglia. The cell suspension was centrifuged, the pellet resuspended in DMEM/F12 medium with 10% FBS and 50 ng mL^−1^ CSF‐1, and the suspension was replated into T25 flasks. After 1–2 h of incubation, nonadherent cells were removed, leaving purified microglia for subsequent experiments.

### CCK‐8 Assay

Cell viability was assessed following the protocols provided by the Cell Counting Kit‐8 (Dojindo Molecular Technologies, Inc., Kumamoto, CK04, Japan). Primary microglia cells were plated in 96‐well plates at a density of 2000 cells per well and incubated in FBS‐free medium for 12 h prior to treatment. Treatments included ASTX029 at concentrations of 1 × 10^−9^, 0.1 × 10^−9^, 0.5 × 10^−9^, 1 × 10^−9^, 5 × 10^−9^ m; cells were incubated with the CCK8 solution for 1.5 h at 37 °C. Absorbance was measured at 450 nm using a BioRad xMark microplate reader. Cell viability was calculated as follows: Cell viability (%) = (absorbance of treated sample/absorbance of control) × 100

### ELISA

Cell culture supernatants were thawed on ice and centrifuged at 1000 rpm for 10 min at 4 °C using a pre‐cooled centrifuge. ELISA kits, including IL‐1β (RK00006), IL‐6 (RK00008), and TNF‐α (RK00027) from Abclon, were brought to room temperature for approximately 30 min to equilibrate reagents and antibodies. The assays were conducted following the manufacturer's instructions. Absorbance at 450 nm was measured using a microplate reader, and the data were recorded for analysis.

### Magnetic‐Activated Cell Sorting (MACS)

RPE‐choroid cells were isolated using magnetic bead sorting. Mice were euthanized, and their eyeballs were enucleated to collect RPE‐choroid tissues, with four choroids pooled per group. The tissues were placed in pre‐chilled RPMI 1640 medium, washed with PBS, and minced into small pieces in digestion buffer containing 20 IU mL^−1^ papain and 200 IU mL^−1^ DNase prepared in Hanks’ balanced salt solution (HBSS). The samples were digested at 37 °C for 30 min, then centrifuged at 1000 rpm for 5 min at room temperature. The supernatant was discarded, and the cell pellet was resuspended in HBSS containing DNase and protease inhibitors to terminate digestion. The cells were further resuspended in 180 µL of pre‐chilled PBS containing 0.5% BSA, followed by the addition of 20 µL of anti‐CD11b microbeads (Miltenyi Biotec, Germany) and incubated at 4 °C for 15 min. The labeled cells were then loaded onto magnetic LS columns placed in a MACS separator. After washing, CD11b‐positive cells were eluted by pushing the plunger through the column, yielding purified cells suitable for downstream PCR analysis.

### Statistical Analysis

Figure legends specify the sample size (*n*) for each analysis. Statistical results were reported as Mean ± standard error of the mean (SEM). To assess data normality, we employed the Shapiro–Wilk test. For normally distributed data, homoscedasticity was assessed using the Brown–Forsythe test within GraphPad Prism 9.4. Multiple comparisons were conducted utilizing either one‐way ANOVA followed by Tukey's post‐hoc analysis or two‐way ANOVA followed by Tukey's post‐hoc analysis, contingent upon the homogeneity of variances. Differences between two groups were assessed using an unpaired two‐tailed Student's *t*‐test. For non‐normally distributed data, unpaired comparisons were performed via the Mann–Whitney test. In the context of the laser‐CNV model, we employed a linear mixed model to simultaneously consider correlations between measurements from the same mouse, under the assumption of exchangeable eyes and correlations for repeated measurements within the same eye (in cases of repeated laser burns).

## Conflict of Interest

The authors declare no conflict of interest.

## Author contributions

Y.Z., J.J., and Y.L. contributed equally to this work. Y.Z., J.J., and Y.L. worked on conceptualization, data curation, formal analysis, methodology, and wrote the original draft. X.D., Y.S., and Q.T. worked on data curation and formal analysis. L.X. and L.C. worked on conceptualization, supervision, funding acquisition, and reviewed and edited the writing. All authors revised and agreed on the final version of the manuscript.

## Supporting information



Supporting Information

## Data Availability

Research data are not shared.
